# Association between Reactive Attachment Disorder/Disinhibited Social Engagement Disorder and Emerging Personality Disorder: A Feasibility Study

**DOI:** 10.1155/2016/5730104

**Published:** 2016-06-06

**Authors:** Khadija Mirza, Gracia Mwimba, Rachel Pritchett, Claire Davidson

**Affiliations:** ^1^Forth Valley Royal Hospital, Larbert FK5 4WR, UK; ^2^Barrhead Health and Care Centre, East Renfrewshire, Glasgow G78 1SW, UK; ^3^University of Glasgow, Health and Wellbeing, Caledonian House, Royal Hospital for Sick Children, Glasgow G3 8SJ, UK

## Abstract

A systematic review of reactive attachment disorder (RAD)/disinhibited social engagement disorder (DSED) in adolescence highlighted that young people with the disorder had indiscriminate friendliness with difficulties in establishing and maintaining stable relationships. Most reported experiences of rejection. We were struck by similarities between the above and features of emergence of personality disorders (EPD). This feasibility study aimed to determine best ways of recruiting and retaining vulnerable young people and the proportion of participants with RAD/DSED who might have emerging borderline personality disorder (EBPD). Participants were referred to the study by their treating clinicians from local mental health teams. Results showed strong association between RAD/DSED and EBPD. Participant characteristics showed high levels of out of home placements, early termination of school careers, suicide attempts, quasipsychotic symptoms, and multiagency involvements. They experienced the project as an opportunity to talk about relationships and reported that they would like more of this in usual clinical contacts. They all agreed to be contacted for future studies. Previous studies have shown that early detection and treatment of emergent personality traits can alter trajectory. Future research will continue to explore these trajectories, explore detection of vulnerability factors, and evaluate interventions.

## 1. Introduction

The study described in this paper has taken root from clinical concerns about the emergence of personality disorders in the young adult population and the possible role of early trauma and attachment.

During the conceptualization stage of the study, a steering group of clinicians realized that there are children and young people who have become homeless and alienated from their families or are living in foster or residential care who may share features of emerging personality disorder. As a result, a group of clinicians called the “emerging personality disorder group” was formed. It is within this group that the project was brought to life.

The importance of early life events in later development is now better understood and associations between early trauma and later social, physical, and mental health problems are now well established [1]. Minnis [2] described the risk that early maltreatment places on young children, conceptualising the overlapping problems, which they are at increased risk of developing (maltreatment associated psychiatric problems, MAPP). The diagnostic manual (DSM-5) describes two disorders that are associated with early maltreatment: reactive attachment disorder (RAD) and disinhibited social engagement disorder (DESD). Although the two share a common aetiology, the former is expressed as an internalising disorder and withdrawn behaviour, while the latter is marked by disinhibition and externalising behaviour. Previously (DSM-IV), these disorders were instead understood as two forms of the same disorder, inhibited and disinhibited reactive attachment disorder. Research has shown that school-age children with reactive attachment disorder (DSM-IV diagnoses) are likely to have complex neurodevelopmental problems [3], but very little is known about how they present in adolescence and their trajectories into adulthood.

Adverse early life events are also known to be associated with personality disorders in adolescence and early adulthood [4]. Investigating personality disorders earlier in their developmental course has been shown to significantly alter the trajectory to full-blown personality disorders [5, 6]. There is a wealth of research currently proving the reliability, validity, and clinical importance of early diagnosis and treatment of emergent personality disorder traits [4, 5, 7]. A significant example of this is the Helping Young People Early Clinic in Melbourne, Australia [5] that has successfully applied these principles and produced good quality evidence base of the effectiveness of early diagnosis and treatment of maladaptive personality traits.

Many adolescents and young people have complex overlapping issues which would benefit from services especially if the intervention was available earlier on which would help alter the trajectory. One reason might be that RAD and DSED are poorly recognised in adolescence and a diagnosis of personality disorder prior to 18 years old is often discouraged and controversial [7].

Children with these disorders are some of the most vulnerable young people in society who, if their trajectory is not altered, are likely to have extremely poor psychosocial outcomes. It is therefore crucial that we better understand the developmental pathways that clinicians suspect might lead from early trauma, through attachment disorders to borderline personality disorder. No study has previously examined the contribution of RAD and DSED toward this developmental trajectory.

Young people with this level of vulnerability are known to be hard to recruit for the purposes of research [5]. This paper describes an initial feasibility/proof of concept study that we hope will lay the groundwork for a larger piece of future research.

Study goals were as follows:

1. To determine the feasibility of conducting research in this population, that is, recruitment, retention, and proportion of eligible young adults.
2. To make a preliminary examination of the proportion of young people with reactive attachment disorder (RAD) and disinhibited social engagement disorder (DESD) who would have emerging borderline personality disorder (EBPD).
3. To profile the young people recruited to the study.

## 2. Material and Methods

NHS West of Scotland ethics committee and NHS Research and Development granted ethics approval. Service management approval was sought from local Child and Adolescent Mental Health Services (CAMHS) and Adult Mental Health Teams (AMHTs) service managers. Participants prior to referral and prior to participation provided informed written consent.

### 2.1. Inclusion Criteria

The inclusion criteria were as follows:

- Written informed consent.
- 15–25 years.
- Indiscriminate friendliness.
- A history of maltreatment/significant trauma not necessary.

### 2.2. Exclusion Criteria

The exclusion criteria were as follows:

- Inability to consent.
- Being in a prison or a young offender in the custody of HM Prison Services or supervised by probation services.

### 2.3. Sample Size

There was no sample size calculation as this was a feasibility study. Using past experience of screening for RAD/DSED, it was agreed that a maximum referral of 50 participants will be sought but also the end of the study will be determined when referrals were enough to make conclusions and answer aims.

#### 2.3.1. Source of Participants

Recruitment took place in a local health board in Scotland, that is, NHS Greater Glasgow and Clyde. Clinical areas approached for recruitment included Child and Adolescent Mental Health Services (CAMHS) teams, Adult Mental Health Teams (AMHTs), Homeless and Trauma team, Looked After and Accommodated (LAAC)/Forensic CAMHS teams.

#### 2.3.2. Participant Characteristics

Participants' age range was between 15.7 and 24.6 years (mean 18.3 years). Out of 24 participants referred to the study by clinicians, 4 were males, that is, 16%. One male gave subsequent written consent but did not meet screening criteria for RAD/DSED.

Hence, the study population, that is, diagnosed with RAD/DSED was solely female.

Participants [8] who met screening criteria had the following informants: 4 parents, 2 grandparents, 1 family friend, and 3 health and care professionals.

The main screening criteria were symptoms of RAD/DSED, which are indiscriminate friendliness or withdrawal and hypervigilance. In adolescence, some features of indiscriminate friendliness could be overdisclosure, risk-taking behaviours including severe self-harm, and overfamiliarity with clinicians.

### 2.4. Recruitment

#### 2.4.1. Phase 1

During a period of 8 months, 13 local CAMHS teams and 3 specialist teams, the Looked After and Accommodated Children's team, Homelessness and Personality Disorder team (mainly targeting young adults) and the Forensic services, were contacted by members of the research team.

#### 2.4.2. Phase 2

To capture the upper age range of the study population, adult mental health teams were approached and the recruitment model was replicated.

There was a research assistant (40% whole time equivalent) and there were 6 senior psychiatric trainees with 4 hours per week each. All clinical teams were given a presentation about the study and a guide to the recruitment. In order to support the recruitment process, a liaison contact clinician from each team was identified; they were contacted fortnightly and a monthly updated newsletter was sent to all recruiting teams. The treating clinicians were asked to briefly explain the purpose and requirements of the study, and, where interest was expressed, a verbal consent was obtained by the clinician in order for the research team to make a telephone contact with the participant. The research team organised a meeting with each participant to obtain written consent and to identify an informant of their choice, for example, a key worker who knew them for more than three months or a primary care giver, for the completion of the CAPA-RAD.

### 2.5. Diagnostic Pathway

#### 2.5.1. Standardised Measures

- *The Observation Schedule for RAD (OSR):* it is a modification of the Waiting Room Observation (WRO), a structured 19-item observation of child behaviour in a clinic waiting room with parents/carers and a stranger [9].
- *Relationship Problems Questionnaire (RPQ)* [10]: it is a 10-item screening instrument for RAD/DSED symptoms.
- *The Child and Adolescent Psychiatric Assessment (CAPA-RAD)* [11]: it is a 30-item semistructured parent/carer interview about RAD symptoms.
- *Maltreatment checklist* [12]: this is a checklist of different forms of abuse. Answers were obtained from case notes, so participants were not asked direct questions about maltreatment.
- *Borderline Personality Questionnaire (BPQ)* [8]: the BPQ is a self-report questionnaire made up of 80 statements, response as true/false.
- *Structured Clinical Interview for the DSM-IV (SCID-II)* [13]: SCID is a semistructured clinical interview for clinical diagnoses. Only the section covering borderline personality disorder was administered in this study.

#### 2.5.2. The Diagnoses of RAD/DSED

 The diagnoses of RAD/DSED were made by the consultant psychiatrists and the research team, based on DSM-5 criteria (the appendix), using the standardised multi-informant assessment package for RAD/DSED (i.e., Relationship Problem Questionnaire, CAPA-RAD, and OSR). This involved the research group together reviewing the patient completed RPQ, the informant completed CAPA-RAD, and the observations made by the researcher, who was unknown to the individual when they first met (OSR). We have used this method of diagnosing RAD in previous research [11] and it has been found to be highly sensitive and specific in discriminating children with RAD from typically developing children. As there have been no UK based studies from which we could develop our diagnostic strategy, we replicated this method [2]. In addition, we spent time, prior to recruitment, thinking how the diagnostic symptoms as described in DSM-5 might manifest in adolescence (e.g., overdisclosure of personal information in adolescence compared to seeking physical closeness to strangers in childhood). DSM-5 suggests that RAD/DSED should only be diagnosed in the presence of a history of “insufficient care,” which was confirmed for all participants via a case note trawl using the “maltreatment checklist.” A maltreatment checklist was conducted by one of the researchers blind to the diagnostic status of the young person by looking at information in the case notes. This was done only in the participants who had both features of RAD/DSED and EBPD. Where the diagnosis was not clear, or data was suggestive but not sufficient, a description of “suspected” diagnosis was used.

#### 2.5.3. The Diagnoses of EPD

 The diagnoses of EPD were made by the research team in participants with a positive diagnosis of RAD/DSED. The diagnosis of EPD was made using results from a screening test, that is, the borderline personality questionnaire (BPQ) followed by the diagnostic test SCID-II for borderline personality disorder only. This diagnostic methodology has successfully been used by Chanen et al. in identifying borderline personality disorder in outpatient youth community [4].

## 3. Results

### 3.1. Recruitment and Retention of Young Adults to This Study

#### 3.1.1. Referring Teams

Out of 24 young people referred, 16 came from the CAMHS teams, 2 from the Adult Community Mental Health teams, and 1 from the Looked After Adolescents, Forensic, and inpatient units.

The recruitment mainly came from clinical researchers directly involved with the study. As a result, 12 out of 16 CAMHS referrals originated from one CAMHS team.

The time taken from obtaining written consent to completion of the data collection ranged from 18 to 250 days (mean 97 days).

### 3.2. Proportion of Eligible Young Adults Who Will Be Willing to Take Part in Such a Study

Following presentations to the team, clinicians would often say that they had young people who would fit study criteria, although this did not always result in the referral. Therefore, it will be difficult to determine the proportion of eligible participants willing to take part in the study.

**Figure 1:**
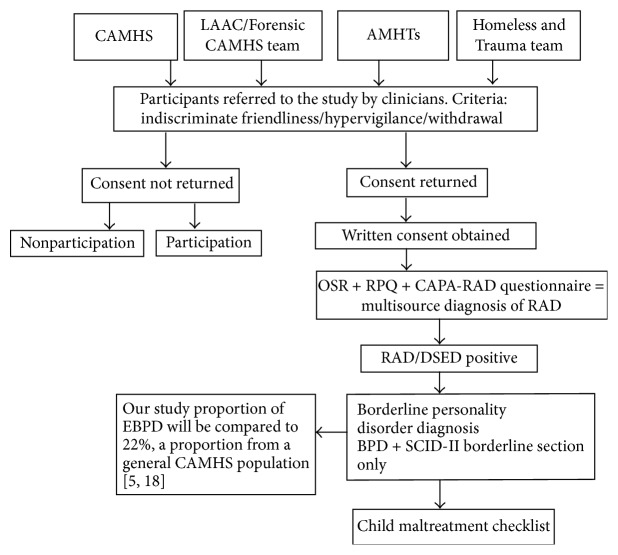
Study flow chart.

Notwithstanding, out of a total of 24 young people approached by their clinicians who potentially met the meeting criteria, 11 did not give consent and 1 was not contactable (Figure 1).

Positive recruitment findings were as follows:

- All the young people who gave consent remained in the study until the end; therefore, there was no dropout.
- The participants also gave positive feedback of being heard and having had an opportunity to talk about their difficulties.
- All the participants agreed to be contacted in the future.
- To our knowledge, there was no worsening of symptoms as a result of the study that would have been reported back to the researchers.

### 3.3. Proportion of Young People with RAD/DSED Meeting Criteria for EBPD

**Figure 2:**
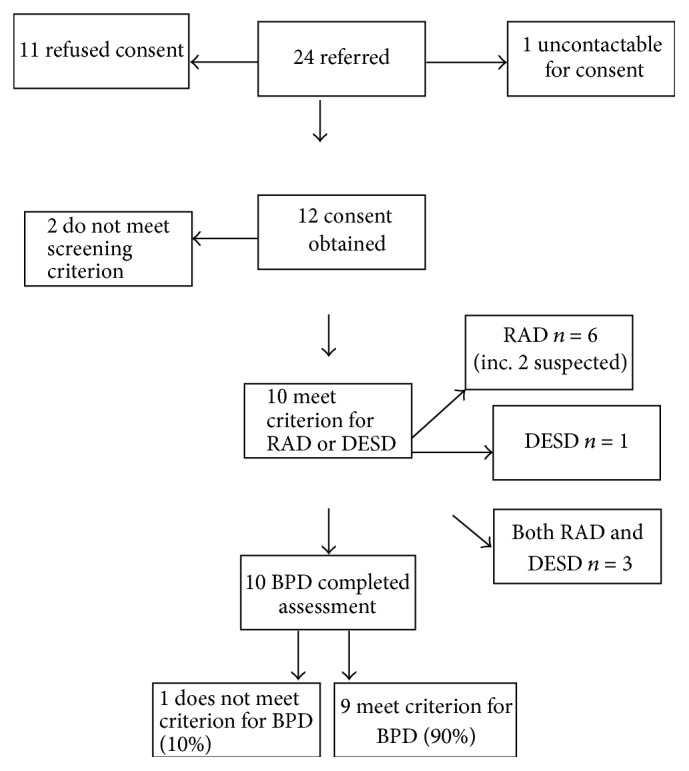
Recruitment, diagnostic procedures, and results.

The literature shows that 22% of outpatients CAMHS population would meet criterion for EBPD, data from Chanen et al. [4]. Our research has shown that, out of 10 young people with RAD/DESD, 9 (i.e., 90%) met the criterion for EBPD. The diagram below shows recruitment and progress throughout the study (Figure 2).

Figure 2 illustrates that 10 participants recruited screened positive for RAD or DSED; therefore, they were eligible for inclusion in the study. Of these, 9 (90%) screened positive for symptoms of BPD.

### 3.4. Other Findings

We found that young people who met criteria for RAD/DSED and EBPD in our study also had the following features:

(i) Four out of ten young people were living at home and 1 was in kinship care. Three out of ten were in supported accommodation; 1 was in homeless accommodation and 1 was sent to a medium secure unit in England. Seven out of ten (70%) were early school leavers (before the age of 16). Nine out of ten (90%) had made at least 1 suicide attempt. Seven out of ten were regularly experiencing perceptual disturbances or pseudohallucinations (72% reported hearing voices; 28% reported both hearing voices and seeing shadows).
(ii) Most participants, who were recruited in the study, were originally referred by one of the CAMHS teams in the area. It was noted within the CAMHS team that young people who met the criterion for the study made 10% (*n* = 12) of the total psychiatric caseload. This clinical population was similar in characteristics with history of trauma representative of our study sample. We noted that, in this clinical population, 6 (50%) young people had confirmed diagnosis of autism and 2 had suspected diagnosis. Another 2 had siblings with a diagnosis of autism spectrum disorder.

### 3.5. The Maltreatment Checklist

All participants had experienced at least one form of abuse. Sexual abuse was recorded as definite (*n* = 6) or suspected (*n* = 2). Physical abuse was reported in 6 participants.

There were also several adverse childhood events reported such as domestic violence, parental separation, and severe parental mental health issues including substance misuse.

### 3.6. Discussion

RAD develops in early or middle childhood as a consequence of significant failures in the care-giving environment. RAD results in social-behavioural issues, remains one of the least evidence-based areas of DSM and ICD nosology, and is associated with multiple maltreatment experiences [14]. Despite the long-standing general agreement that personality disorders, (PD), stem from childhood and adolescents, diagnosing PDs in youth is ever controversial than in adults. Borderline personality disorder is a severe mental disorder that is characterized by a pervasive pattern of impulsivity, emotional instability, interpersonal dysfunction, and disturbed self-image. The results from this study are suggestible of the role of early trauma and its association with the development of RAD, which latterly attributes to the behavioural and personality issues.

#### 3.6.1. The Feasibility of Recruiting and Retaining Vulnerable Young People within a Research Study

Young people with significant difficulties in establishing and maintaining relationships often present as a recruitment challenge; this has been reported in groups such as homeless population. We realized from this study that it is challenging and difficult to reach population group, albeit not impossible to recruit [15].

There is little clinical evidence that explores the challenges associated with the recruitment process. Ejiogu et al. [16] looked at the factors facilitating the recruitment and retention of participants in community studies. The study identified clear communication of the research hypothesis and explanation of the obvious direct benefit to the participants as the key findings not only to improve the recruitment but also to enhance the retention in the study. In addition, we faced challenges directly related to the vulnerability of this client group, as many clinicians were concerned about referring such individuals to a research study. Concerns raised included the ability of the young person to cope with the perceived demands of the research study, that is, meeting individuals who they were unfamiliar with and discussing sensitive areas of their lives such as relationships and how they felt about themselves.

In order to reassure clinicians and to best support the individual, we ensured that no aspect of their history was discussed. We also ensured that the clinician sought written consent for initial contact in order to explain the study hypothesis prior to the first meeting. This provided the young person with the option to change their mind about their participation once they had better understanding of the study, which did prove to be the case in some. Some individuals encountered crisis since the referral was made; therefore, the initial phone call enabled assessment of the young person's eligibility. The mental state of the participant was also closely monitored during the meetings with the researcher and any raised concerns were reported back to the referring clinician. It is really important to note that, of the 10 participants retained within the study, no negative feedback was given with regard to their experiences of research when asked to share their experiences. Many individuals reported experience of “being heard,” “helping others too,” and “doing something worthwhile.”

During the recruitment process, referring clinicians sought the consent from the participants to be contacted by the researcher within 2 weeks, if the consent form had not been returned to the researcher. Of the 10 participants retained in the study, only 2 actually returned the consent form prior to researcher contact. We also found that participants were more likely to attend meetings if they were arranged immediately after the scheduled appointment with their referring clinician. This possibly could have supported individuals, who have had difficulties with organisation due to their underlying chaotic lifestyles. Finally, from the participant feedback, we found that the majority of the individuals preferred to be contacted about such meetings via text rather than direct telephone (see Table 1). This could be due to the fact that they found interaction with unfamiliar person much easier over the text rather than the phone call. This could be attributed to the lack of tangible interaction that is associated with an unknown person over the phone call.

#### 3.6.2. Preliminary Examination of the Proportion of Young People with Reactive Attachment Disorder Who Would Have Emerging Personality Disorder

In this high-risk sample, there seems to be a strong association between maltreatment associated disorders and emerging personality disorder. The disproportionate gender variance with female dominance in the study remains inconclusive, as there is limited data to suggest gender difference in BPD. In the community-based sample of children and adolescents, the prevalence of the BPD was 11% at 9–19 years and 7-8% at 11–21 years and this disorder was more common in girls than boys. However, the results of the previous studies suggest that females and males with BPD may display impulsivity differently. Males may tend to externalise, while women may be more likely to internalise their impulsivity, which is reflected in higher incidence of deliberate self-harm in females consistent with the findings from our study [17].

#### 3.6.3. Discussion of Other Findings

Literature suggests that there is higher incidence of comorbidity associated with borderline personality disorder. This includes mood disorder, anxiety disorder, psychotic symptoms, and substance misuse compared to the general population [2, 18, 19]. Literature further speculates that maltreatment associated psychiatric problems such as RAD/DSED may be an example of environmentally triggered neurodevelopmental presentations. These findings are shared in our study group; however, we will be cautious about interpreting them due to our small population group. They still represent a valuable research query about the possibility of a link between maltreatment associated psychiatric illnesses and neurodevelopmental difficulties [2, 19].

#### 3.6.4. Limitations

There were some limitations to the study: first being the challenges associated with the recruitment process despite the valiant efforts in establishing contact with the clinicians. During the entire recruitment process, only 1 clinician, who was not directly affiliated with the study, was able to refer a patient. The rest as mentioned before came from the clinicians involved with the study. When involved in treatment, the patients challenge their clinician's empathic capacities, standard repertoire of skills and beliefs about what is supposed to happen in the treatment process [20], yielding challenges in recruitment.

The other limitation is the lack of understanding of the factors that precluded the patients from participation. This was yielded by the fact that 50% of the patients changed their mind during the period of their verbal consent to taking formal consent, which could be speculated to be a particular aspect of this group of patients who initially consented for telephone contact with their treating clinician but latterly refused to participate. As there seems to be an imperative role of neglect in this group of patients, it seems highly likely that the informants might not be privy to the experience of the abuse, as literature suggests that there could be underreporting of abuse [16, 17, 21].

Although most clinicians will agree with our finding of a strong association between RAD/DSED and EBPD, we make this conclusion with great caution due to our small study sample. A longitudinal study will be extremely valuable to further decipher this association.

## 4. Conclusions

Maltreatment endured in early life in the form of abuse and social neglect is often implicated in the development of personality disorders in adults, but little empirical research has investigated the role of childhood maltreatment in the development of the personality disorders. It is envisaged that a better understanding of the earlier life maltreatment and its impact will assist develop more effective treatments in the form of early intervention to alter their trajectory [21]. In view of some of our preliminary findings, we explored the link between neurodevelopmental disorders and trajectory of enduring mental illness. We also looked at the vulnerability risk factors, including attachment difficulties, adverse childhood events, and neurodevelopmental disorders, which could be predicted in school and preschool children to allow early interventions and ultimately a healthier trajectory.

### 4.1. Future Research

Future research areas would include longitudinal studies, following up young people with a history of maltreatment to ascertain emergence of severe and enduring mental illnesses. Another area will involve the evaluation of treatment modalities with specific interventions and the role of relevant services including at the interface between CAMHS and general adult psychiatry. Research suggests considerable flexibility and malleability of borderline personality disorder traits in youth, making this a key developmental period, most suitable for intervention [4].

## Figures and Tables

**Table 1:** Views of the participants regarding the best way of future contact.

ID	What are the best ways to contact, including maintain contact, with young people? (We are interested particularly in your views of the use of social media)
(1)	“…I think the best way to contact people is to phone them directly. Both now and in the future. Ask permission to contact them by phone. Also ask permission to text, as some people prefer text. Facebook is a good widespread marketing tool, preference for twitter… Can only say so much and straight to the point. The thing with twitter is that if they are not following they cannot get in contact directly, so would need to make sure people follow first.”

(2)	“…Text is the best way. I use email, but I'm probably different that way. In a text you get the chance to think about what to say, may not want to respond. Young people often won't answer calls. Facebook is a preference to twitter, although have both? If a link on Facebook you can go straight onto the page. Best thing is to keep the page simple. Other options for advertising the study are the young scots magazine and providing information directly to the workers in supported accommodation settings.”

(3)	“I avoid phone calls, use social media more. I use Facebook and twitter equally, Facebook is lengthy but twitter to the point.”

(4)	“…Telephone. Texts after to help give info. Some people won't answer unknown numbers. Facebook is the most frequent way of sharing info (doesn't use often but others do). People have large numbers of friends and can share among friends.” “I do like to look through the adverts on Facebook.” “So may be interested if I saw the study and especially if other friends were sharing it.”

(5)	“I use Facebook and snapchat but wouldn't like research team to contact me through those.” “I would only like to be contacted through the service team and by phone.”

(7)	“I spend hours on the phone. I use instagram mainly but it is to share photos and videos. Otherwise twitter- lot of people on it to look at celebrities but people tend to use Facebook more to share what are doing. A study feed would be useful to keep up to date with findings. Overall I would prefer text to keep in contact. Also suggests that face to face is best and suggests us coming out to schools to speak to people directly.”

(8)	I use Social media, webpage- specific page for the site. I Like Facebook- can be used to informally have an official page; also use Facebook to link to site and twitter. I would personally go on if saw link, even out of boredom/nosey. Changes and progress = website, personal contact = phone or text, not using email anymore.

(9)	“Phone is best.” “I ignore anyone I don't know on Facebook. I don't use email. I use instagram and snapchat but not twitter.” For research study I would just want to be phoned.

(10)	…Facebook and occasionally I use email, but not often. I don't use twitter or anything like that.

(12)	“Phoning is the best way to contact.” Instagram is the only social media I use- just photos, not writing to folk. I used to use Facebook but not anymore. I don't like Facebook; think it's really unhealthy with bullying and fake pages about people. If you complain it takes a while before stuff gets removed. People can also get addicted and can cause competitiveness.

(15)	“I use Facebook and instagram to talk to friends, I don't use twitter.” “My opinion is that phoning is the best way to contact young person for research.”

(16)	“Please send letters to my physical home address only”

## Appendix

### DSM-5 Criteria for Reactive Attachment Disorder (RAD)

The* DSM-5* gives the following criteria for Reactive Attachment Disorder:

- A consistent pattern of inhibited, emotionally withdrawn behaviour toward adult caregivers, manifested by both of the following:
  The child rarely or minimally seeks comfort when distressed.
  The child rarely or minimally responds to comfort when distressed.
- A persistent social or emotional disturbance characterized by at least two of the following:
  Minimal social and emotional responsiveness to others.
  Limited positive affect.
  Episodes of unexplained irritability, sadness, or fearfulness that are evident even during nonthreatening interactions with adult caregivers.
- The child has experienced a pattern of extremes of insufficient care as evidenced by at least one of the following:
  Social neglect or deprivation in the form of persistent lack of having basic emotional needs for comfort, stimulation, and affection met by caring adults.
  Repeated changes of primary caregivers that limit opportunities to form stable attachments (e.g., frequent changes in foster care).
  Rearing in unusual settings that severely limit opportunities to form selective attachments (e.g., institutions with high child to caregiver ratios).
- The care in Criterion (C) is presumed to be responsible for the disturbed behaviour in Criterion (A) (e.g., the disturbances in Criterion (A) began following the lack of adequate care in Criterion (C)).
- The criteria are not met for autism spectrum disorder.
- The disturbance is evident before age 5 years.
- The child has a developmental age of at least nine months.

*Specify If Persistent*. The disorder has been present for more than 12 months.

*Specify Current Severity*. Reactive Attachment Disorder is specified as severe when a child exhibits all symptoms of the disorder, with each symptom manifesting at relatively high levels.
